# Kinetic and Isothermal Investigations on the Use of Low Cost Coconut Fiber-Polyaniline Composites for the Removal of Chromium from Wastewater

**DOI:** 10.3390/polym14204264

**Published:** 2022-10-11

**Authors:** Stuti Jha, Rama Gaur, Syed Shahabuddin, Irfan Ahmad, Nanthini Sridewi

**Affiliations:** 1 Department of Chemistry, School of Technology, Pandit Deendayal Energy University, Knowledge Corridor, Raysan, Gandhinagar 382426, Gujarat, India; 2 Department of Clinical Laboratory Sciences, College of Applied Medical Sciences, King Khalid University, Abha 61421, Saudi Arabia; 3 Department of Maritime Science and Technology, Faculty of Defence Science and Technology, National Defence University of Malaysia, Kuala Lumpur 57000, Malaysia

**Keywords:** adsorption, heavy metals, environmental remediation, wastewater, polyaniline, coconut fiber

## Abstract

Pollution due to various heavy metals is increasing at an alarming rate. Removal of hexavalent chromium from the environment is a significant and challenging issue due to its toxic effects on the ecosystem. Development of a low-cost adsorbent with better adsorption efficiency is presently required. In this study, waste coconut fibers (CF) were used to prepare its composite with polyaniline (PANI) via in-situ oxidation. The obtained composites with varying loading of PANI (15, 25, 50, and 75% *w*/*w*) were characterized by FE-SEM, TGA, and FTIR spectroscopy. The prepared composites were evaluated for their adsorption performance for removal of Cr(VI). It was concluded that the composite with 50% *w*/*w* polyaniline loading on coconut fiber exhibited a maximum adsorption efficiency of 93.11% in 30 min. The effect of pH, dosage, and concentration of the aqueous solution of chromium on the Cr(VI) adsorption efficiency of the composite was also studied. From the optimization studies it was observed that the absorbents exhibited the best adsorption response for Cr(VI) removal with 0.25 mg/mL adsorbent at pH 4, in 30 min. The effect of pH, dosage, and concentration of the aqueous solution of chromium on the Cr(VI) adsorption efficiency of the composite was also studied. This study highlights the application of low-cost adsorbent as a potential candidate for the removal of hexavalent chromium. A detailed study on the adsorption kinetics and isothermal analysis was conducted for the removal of Cr(VI) from aqueous solution using coconut fiber-polyaniline composite. From the kinetic investigation, the adsorption was found to follow the pseudo second order model. The data obtained were best fitted to the Elovich model confirming the chemisorption of the Cr(VI) on coconut polymer composites. The analysis of the isothermal models indicated monolayer adsorption based on the Langmuir adsorption model.

## 1. Introduction

Heavy metal pollution is a prime concern for the society due to their toxicity, persistent nature and bioaccumulation in the environment [1]. Heavy metals are metals with densities greater than 5 gm/cm^3^ and atomic numbers greater than 20 [2].Such metals pose a serious threat to human, plant, and animal health. Because of their toxicity, heavy metal removal should be considered. Heavy metals are omnipresent in the environment, the concentration of which is increasing due to modern day urbanization and industrialization [3].Heavy metals include Cr, Hg, Pb, Co, Ni, Cu, Zn, Sn, and Cd, etc. Chromium is a naturally occurring element with valency ranging from II to VI [4]. The main oxidation state of chromium is III and VI. When chromium is released into the environment due to various activities, it is mainly in its hexavalent form [5]. The hexavalent state of chromium is more stable and mobile than its trivalent state. Cr(VI) is a common contaminant in many environmental systems as it is widely used in various processes such as in dyes and pigments, leather tanning, chrome plating, etc. [6,7,8]. Many methods are implemented for the removal of heavy metal like adsorption, electro dialysis, ion-exchange, reverse osmosis, and ultra-filtration, etc. [9,10,11,12,13,14]. Among all the methods used for heavy metal remediation, adsorption is the most widely adapted method [15]. The adsorption method involves a simple set up and has higher performance efficiency. It is a regenerative and a cost-effective method making it the most feasible method for heavy metal removal [16]. Many low-cost adsorbents have been used by researchers for the adsorption study of chromium. Several studies using agricultural wastes such as banana peels, citrus limetta peels, coconut husk, potato peels, palm pressed fibers, and sawdust, etc. as an adsorbent for the treatment of chromium have been reported [17,18,19,20,21]. However, their efficiency is limited and can be modified by combining them with other suitable materials. This awakens the necessity of development of new or modification of the already used adsorbents for effective Cr(VI) removal.

Recently, conducting polymers have attracted a lot of attention in pollutant adsorption due to their properties such as special morphologies, functional groups and simple synthetic procedure [22]. They have the ability to remove heavy metals through complexation and ion-exchange mechanism [23]. Polyaniline (PANI) is a polymer which has been explored in recent years for its potential as a heavy metal adsorbent. PANI, a conducting polymer with terminal amine (–NH_2_) group has excellent properties such as high surface area, adjustable surface chemistry, desirable pore size distribution, rigidity, and economical regeneration [24]. Apart from PANI, PANI-based composites have also been studied for their application in heavy metal adsorption. PANI-based composites offer added advantages such as higher surface area, higher dispersibility, enhanced adsorption performance, and combined properties of the polymer and the substrate [22,25]. Dutta et al. (2021) synthesized polyaniline-polypyrrole copolymer coated green rice husk ash and investigated its potential for Cr(VI) removal [26]. PANI-jute fiber was synthesized by Kumar et al. (2008) for removal of hexavalent chromium from wastewater [27]. PANI-magnetic mesopores silica composite was used an adsorbent for chromium adsorption by Tang et al. (2014) [28].Hexavalent chromium was adsorbed on the surface of PANI-rice husk nanocomposite by Ghorbani et al. (2011) [29]. Lei et al. has reported the use of PANI-magnetic chitosan composite for the removal of hexavalent chromium [30]. Rahmi et al. reported the use of using chitosan based composites chitosan for the removal of Cd(II) from its aqueous solution [31,32]. Cr(VI) was adsorbed using gelatine composites in a study reported by Marciano et al. [33]. From the literature review, it was inferred that all the similar studies have either reported the use of large amount of adsorbents (1 to 125 g/L) for the removal of contaminants or a more time consuming process (up to 5 to 6 h). In addition, the removal efficiency is also less in comparison to the present study. Thus, it was observed that the present study offers certain advantages such as using low-cost adsorbent, less adsorbent dosage, and high efficiency in less time. In this study, coconut fibers have been used as a substrate and PANI has been dispersed on its surface using in-situ polymerization. Agricultural waste such as coconut fiber has advantages over other substrates such as being easy collectable and available with less or no cost. Moreover, it involves simple processing steps (washing, drying, sieving) and thus reduces energy and production cost. This study aims to focus on the synthesis of PANI-coconut fiber and its application for Cr(VI) removal. It also reports the effect of various parameters such as pH, adsorbent dosage and concentration of Cr on the adsorption capacity of the composite. A detailed investigation on the kinetic aspects and adsorption isotherm has been performed.

## 2. Materials and Methods

### 2.1. Materials

In this study, coconut shells were collected from the local market of Gandhinagar, Gujarat, India. Potassium dichromate (K_2_Cr_2_O_7_) (SRL (Ahemdabad, Gujarat, India) AR grade, extrapure, 99.9%) was used as the source of Cr(VI). Aniline used in this process was purified using distillation process prior use. All the chemicals used for the preparation of composites including aniline, ammonium persulfate (APS) (Sigma Aldrich (Ahemdabad, Gujarat, India), reagent grade, 98%), HCl (Finar (Ahemdabad, Gujarat, India), AR grade, 37% purity) were used as received. All the dilutions performed in this study were carried out using milli pore water.

#### 2.1.1. Pre-Treatment of Coconut Fibers

The collected coconut shells were separated into coconut fibers (CF) and washed to remove dirt. The coconut fibers were then dried under shade. The dried coconut fibers were cut into pieces before grinding them to make a fine powder. The obtained coconut fiber powder was sieved to obtain particles of uniform size (≤ 75 microns).

#### 2.1.2. Synthesis of Polyaniline (PANI) and Polyaniline-Coconut Fiber (PANI-CF) Composites

The composites were prepared with different *w*/*w*% loading of PANI on CF. PANI was prepared by in-situ oxidation method. For this process, a solution of aniline in 1 M HCl was prepared. Another solution of ammonium persulphate (APS) dissolved in 1 M HCl was added dropwise with constant stirring for 2–3 h. The reaction temperature was maintained between 0 to 5 °C. Subsequently, the reaction mixture was filtered and washed with 0.5 M HCl until the filtrate became colorless and then with deionized water until the filtrate became neutral. Then, the obtained PANI was dried in vacuum oven at 80°C overnight. The composites with varying loading of PANI were prepared using a similar approach. The schematic flow of both the processes is shown in Figure S1a,b (Supplementary Materials). The digital images of the prepared composites are shown in Figure S1c (Supplementary Materials).

Composites were prepared with different *w*/*w*% loading of PANI. The samples were coded as CFC15, CFC25, CFC50 and CFC75 for 15%, 25%, 50% and 75% PANI, respectively. For preparation of all the composites, the starting weight of CF was kept 0.5 gm and the weight of PANI was varied. Rest all steps were similar to the synthesis of PANI. Depending on the composition of PANI, the prepared samples were named CFC15, CFC25, CFC50, and CFC75 as listed in Table 1.

## 3. Characterization

All the samples in the present study were analyzed for their functional groups, morphology and thermal stability using different characterization techniques such as Fourier-transform infrared spectroscopy (FT-IR), Field Emission-Scanning Electron Microscopy (FE-SEM), and Thermal gravimetric analyzer (TGA). The Fourier transform- infrared spectra of the samples were recorded using FT-IR spectrometer Perkin Elmer (Mumbai, Maharashtra, India), spectrum 2 model in ATR mode in a scan range of 400 to 4000 cm^−1^. The FE-SEM images of the samples were taken in Zeiss ultra 55 model (Bangalore, Karnataka, India) at acceleration voltage of 5.00 kV. For FE-SEM analysis the samples were sprinkled on clean aluminum stub over conducting carbon tape. The samples on aluminum stubs were coated with a thin gold layer using LEICA EM ACE200 (Wetzlar, Hesse, Germany) to make them conductive. The thermal stabilities of the samples were analyzed using Thermal gravimetric analyzer (Eltra sthermostep, (Hyderabad, Telangana, India) with a heating rate of 10 °C/min with temperature range 200 to 950 °C in O_2_ atmosphere. The concentration of the aqueous solution of chromium during adsorption study was monitored using LABINDIA analytical model 3000^+^Ultraviolet-visible (UV-Vis) Spectrometer (Ahemdabad, Gujarat, India), in absorbance scan mode in the range of 200–800 nm.

## 4. Adsorption Studies

In the present study, adsorption of Cr(VI)in aqueous solution using CFC15, CFC25, CFC50, CFC75 and PANI was carried out at room temperature in batch mode. For the adsorption studies, a test solution of Cr(VI) (10 ppm) was prepared and used for adsorption. For the preparation of 10 ppm solution, 0.02828 gm of potassium dichromate was dissolved in 1 L of water. For the detailed study for the adsorption of chromium in aqueous solution, different sets of experiment were performed. The effect of different parameters such as dosage of adsorbent, concentration of chromium solution and pH were explored for adsorption of Cr(VI) in aqueous solution. In a typical adsorption experiment, 0.25 mg/mL of adsorbent was added to aqueous solution of chromium (10 ppm). The mixture was sonicated for uniform dispersion of the adsorbent. The suspension with the adsorbent was kept for constant stirring for 30 min for shaking on a mechanical shaker. After completion of 30 min, the solution was centrifuged to remove the adsorbent and the solution was analyzed using UV-Vis spectrometer. The analysis of the final concentration of chromium was done by monitoring the absorbance at a wavelength of 352 nm. The kinetics of the adsorption studies were monitored by taking aliquots at regular interval of time. The supernatant was analyzed using UV-Vis spectrometer. For investigating the effect of dosage, similar studies were conducted with amount of adsorbent from 0.05 mg/mL to 1 mg/mL in aqueous solution of chromium (10 ppm). To explore the effect of pH, the adsorption studies were conducted at different pH2, 4, 7, and 9. The acidic pH was adjusted using 0.1 M HCl while the basic pH was adjusted using 0.1 M NaOH. The spectral data obtained were analyzed for each sample and fitted into different kinetic and isothermal models to determine the nature of the process. The % removal and adsorption at equilibrium were calculated using the following formula,

(1) $$\;\%\;\mathrm{removal}=\frac{C_{1\;}-C_{2}}{C_{1}}\times 100$$

The adsorption at equilibrium will be calculated using,

(2) $$Q_{e}=V\left(C_{1}-C_{2}\right)÷M$$

*Q_e_* = amount of adsorption at equilibrium (mmol/g);

*V* = volume of heavy metal solution taken (ml);

*M* = quantity of adsorbent added (mg);

*C*_1_ and *C*_2_ (mg/L)refer to the heavy metal concentration before and after adsorption respectively at the λ_max_.

The kinetic data was fitted to different kinetic models such as first order, second order, pseudo first, pseudo second order, Elovich, and intra-particle diffusion. For adsorption isotherm analysis, experiments were performed using different concentration of chromium solution from 10 to 50 ppm. To each solution, 0.25 mg/mL of adsorbent was added. The adsorption process was carried out for 30 min following the same steps as mentioned above. The data collected was analyzed using Langmuir, Freundlich, and Temkin isotherm models. All the experimental data in terms of concentration and %removal were fitted to standard isotherm models using Origin pro 2021.

## 5. Results and Discussions

The synthesized samples were analyzed for structural, thermal, morphological analysis. The characterization and adsorption results are as follows.

### 5.1. Characterization of the Samples

#### 5.1.1. Thermal Stability Analysis

**Figure 1:**
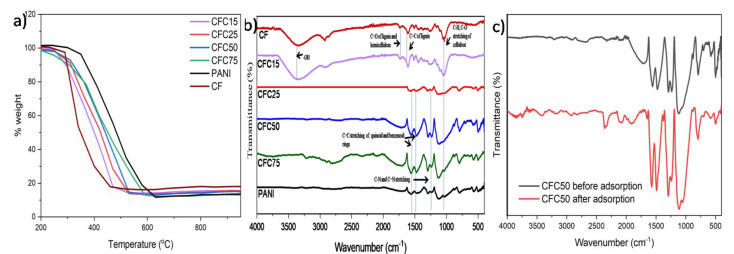
(**a**)TGA curve of CF, PANI, CFC15, CFC25, CFC50, and CFC75, (**b**)FTIR spectra of CF, PANI, and its composites, and (**c**) CFC50 before and after the adsorption of Cr(VI).

Figure 1a shows the thermogram of CF, PANI and its composites. In general, a weight loss is observed with increase in the temperature for the raw materials and composites used in this study. From the TGA curve of CF, it was interpreted that the decomposition temperature was 343 °C where it suffered maximum weight loss (79.5%). On the other hand, the TGA curve of PANI showed that the weight loss was maximum (84.8%) at temperature 485 °C. The loading of PANI on CF lead to increased stability of composites as evident from TGA analysis. An increase in T_d_ is observed in composites with varying % of PANI as compared to CF. The decomposition temperature obtained from TGA has been listed in the Table 2. Thus, preparation of composites enables us to develop more thermally stable materials which make them a suitable candidate for adsorption process.

#### 5.1.2. Functional Group Analysis

Since adsorption is a surface phenomenon it becomes crucial to analyze the functional group present on the adsorbent surface. The understanding of functional groups helps to explore the adsorption mechanism and the nature of the process. Figure 1b shows the IR spectra of CF, PANI, and the prepared composites. In the IR spectrum of CF, the characteristic bands at around 3400 cm^−1^ is assigned to –OH stretching, the peaks at 1750 and 1240 cm^−1^ are attributed to C=O stretching of lignin and hemicellulose and C–H, C–O stretching of cellulose [26,34].Other peaks at 1614 cm^−1^ is for C=C of lignin and 1440 cm^−1^corresponds to C–H vibration. Similarly in a study investigating thermally treated wood samples, Cheng et al. also observed C=C stretching vibrations at 1603 cm^−1^ [35]_._IR peaks observed in the spectrum of PANI at 1568 and 1489 cm^−1^correspond to C=C stretching of quinoid and benzenoid rings, respectively [36]. The peak at 1292 cm^−1^ correspond to C–N and C=N stretching. The peak for out-plane and in-plane C–H bonding is observed at 795 and 1106 cm^−1^ [37,38,39,40]. The oxygen and nitrogen containing functional groups offer potential binding sites for the adsorption of heavy metals. These functional groups tend to increase the cation-exchange capacity of the material by creating electron donor centers in the aqueous medium [41,42]. For the confirmation of the adsorption of Cr(VI) on the surface of CFC50, FTIR spectrum of CFC50 after chromium adsorption was also recorded (Figure 1c). A shift in IR peak of CFC50 at 1561 cm^−1^ corresponding to C=C stretching of quinoid to 1572 cm^−1^ was observed after the adsorption depicting the adsorption of chromium on the surface of CFC50. The intensity of peakat 1292 cm^−1^ corresponding to C–N and C=N stretching also changes before and after adsorption showing the involvement of nitrogen containing functional group in the adsorption process. In the IR spectrum of CFC50 after adsorption, a new peak at 1051 cm^−1^ can be observed. Similar results have been reported by Dula et al., Solgi et al., and Shooto et al. [43,44,45].

#### 5.1.3. Morphological Analysis

**Figure 2:**
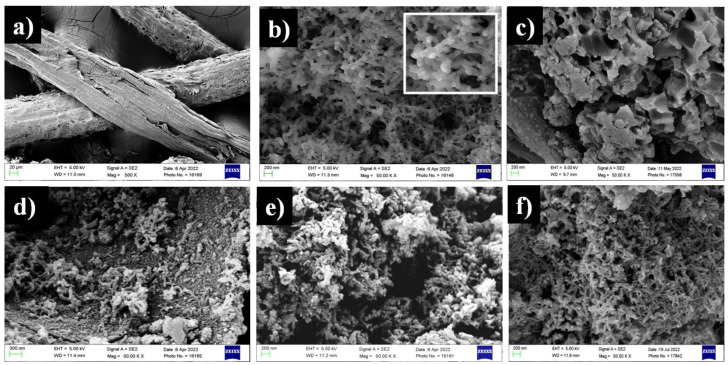
FE-SEM images of (**a**) CF, (**b**) PANI, (**c**) CFC15, (**d**) CFC25, (**e**) CFC50, and (**f**) CFC75. (inset of (**b**) shows the higher resolution image of PANI depicting the rod like shape with agglomerates).

Figure 2 shows the FE-SEM image of CF, PANI, and their composites. The FE-SEM image of CF clearly shows the presence of fibrous shape morphology (Figure 2a) while PANI exhibits a rod like shape with agglomerates as shown in Figure 2b. Similar fibrous morphology for CF and rod-like shape for PANI has been reported by Dutra et al. and Martina et al. respectively [36,46]. The FE-SEM images of composites shown in Figure 2c–f depict dispersion of rod like particles over CF confirming the formation of CF-PANI composites. Additionally, as the concentration of PANI increases in the composites, the amount of rod like particles dispersed on the CF increases which confirm the proper loading of PANI on CF.

### 5.2. Adsorption Study

**Figure 3:**
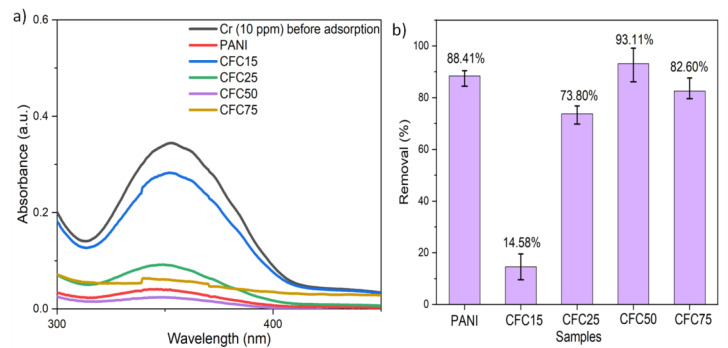
(**a**) UV-Vis spectra depicting the adsorption performance of the prepared samples (**b**) % adsorption of prepared samples for Chromium adsorption (pH = 6, adsorbent dosage = 0.25 mg/mL, Cr concentration = 10 ppm, contact time = 30 min).

The prepared composites namely CFC15, CFC25, CFC50, CFC75 and PANI were evaluated for Cr(VI) adsorption in aqueous solution. Figure 3a shows the UV spectra of the comparative performance/adsorption ability of CF, PANI, and composites. The results indicated a drastic reduction in the intensity of absorption peak at λ_max_of 352 nm confirming the removal of Cr(VI) from aqueous solution. The adsorption efficiency of PANI, CFC15, CFC25, CFC50, and CFC75 was found to be 88.41%, 14.58%, 73.80%, 93.11%, and 82.60%, respectively as represented in Figure 3b. From the adsorption results, it was inferred that the preparation of CF-PANI composite (CFC50) showed enhanced adsorption efficiency as compared with PANI. This is attributed to the synergic effect of CF and PANI in the composites, for improved adsorption of Cr(VI) in aqueous solution. Thus, the preparation of composites of PANI with CF is a cost-effective and sustainable method for the removal of Cr(VI). An improved performance as compared with a pristine PANI sample was observed for smaller amount of PANI when dispersed over CF. Additionally, by further increasing the amount of PANI on CF (CFC75) the adsorption efficiency decreased from 93.11% to 82.60%. It is proposed that coconut fibers act as a support and promote uniform dispersion of PANI over its surface. The poor efficiency of CFC15 is attributed to smaller loading amounts of PANI and its non-uniform dispersion. An increase in the performance was observed with increased loading of PANI until 50%. The sudden decrease in efficiency for CFC75 is due to the agglomeration of particles and unavailability of more active surface sites. From the studies we can conclude that the development of CFC50 reduces the cost and results in improved performance as compared with PANI.

### 5.3. Kinetic Studies

The kinetics of adsorption of Cr(VI)in aqueous solution were monitored at different time intervals. Figure S2a–e (Supplementary Materials) shows the UV-Vis spectrum for the kinetic studies for different adsorbents used. A continuous decrease in absorbance indicates the removal of Cr(VI) from aqueous solution. Figure S2f (Supplementary Materials) shows the absorbance vs. time graph for CF, PANI, and its composites. From the results, it was concluded that CFC15 and CFC75 showed desorption of Cr(VI) after 10 min, while no desorption could be seen in cases of PANI, CFC25, and CFC50.

The kinetic data obtained from the UV-Vis spectra was then analyzed using a different kinetics model to better understanding of the process. The kinetic data was fitted into Pseudo first order (PFO), first order (FO), pseudo second order (PSO), second order (SO), Elovich model, and intraparticle diffusion corresponding to the same concentration of Cr(VI)aqueous solution (10 ppm). Equations of the kinetic models studied in the present work are as mentioned in Table S1 (Supplementary Materials).

**Figure 4:**
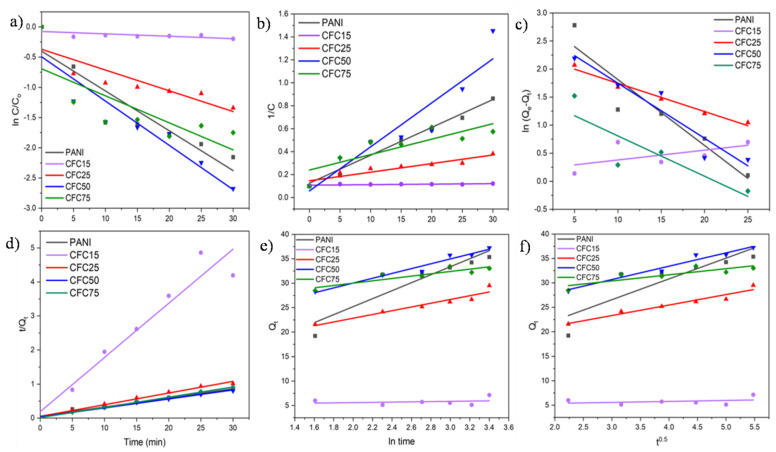
Kinetic models for the adsorption of Cr(VI) onto the prepared samples (**a**) First order, (**b**) Second order, (**c**) Pseudo first order, (**d**) Pseudo second order, (**e**) Elovich, (**f**) Intra-particle diffusion, (pH = 6, adsorbent dosage = 0.25 mg/mL, Cr concentration = 10 ppm, total contact time = 30 min).

Figure 4 shows the graph plotted to understand the kinetics for different models. The parameters for the linear fitting analysis such as R^2^, rate constant (K), etc. are listed in Table 3. From the high R^2^ values, the pseudo second order kinetic model was found to be the best fitted for the present adsorption study. The Elovich and Intraparticle diffusion models were also analyzed to understand the mechanism of adsorption. The intraparticle diffusion model states that the adsorption process is controlled by either film diffusion, pore diffusion, or surface diffusion or their combination [47].Ofomaja et al. reported that the adsorption of chromium by magnetite coated biomass followed the intraparticle diffusion model [48]. The Elovich model is a widely adapted in adsorption kinetics, used to describe chemical adsorption [49]. The value of R^2^ indicated that the Elovich model was better suited to understand the mechanism. Elovich models hints towards chemisorption and is more suited for the heterogeneous surface of the adsorbent [50]. Similar results were reported byAworanti et al.for the adsorption of Cr(VI) by sawdust derived activated carbon [51].

### 5.4. Isotherm Study

The isothermal analysis was performed for the composite sample CFC50 as it was found to be the best adsorbent for the removal of Cr(VI) among all the samples. For isothermal analysis, different concentrations of chromium in its aqueous solution were used. The experimental data obtained were plotted in the form of Q_e_ versus C_e_ (concentration at equilibrium) to study the Langmuir, Freundlich, and Temkin adsorption isotherm [52,53]. The data were fitted with the non-linear form of all the isotherms shown in Figure S3 (Supplementary Materials). The linear and non-linear equations of the isotherms are listed in Table S2 (Supplementary Materials). The comparative analysis of R^2^value for all the isotherm models as shown in Table 4 indicated that the data were best fitted in Langmuir isotherm equation. Hence it can be concluded that the adsorption process of Cr(VI) using CFC50 follows the Langmuir model. From the analysis it is inferred that the adsorption occurs at a specific homogeneous site and is limited to one layer. The isotherm assumes that there is a formation of a monolayer adsorbate on the outer surface of adsorbent. After the formation of this layer no further adsorption takes place. Piccin et al. (2011) and Dada et al. (2012) reported similar results for the adsorption of food dye and Zn^+2^ by chitosan and rice husk, respectively. In both the cases the adsorption followed the Langmuir adsorption isotherm [53,54].

### 5.5. Effect of Adsorbent Dosage on Chromium Adsorption

**Figure 5:**
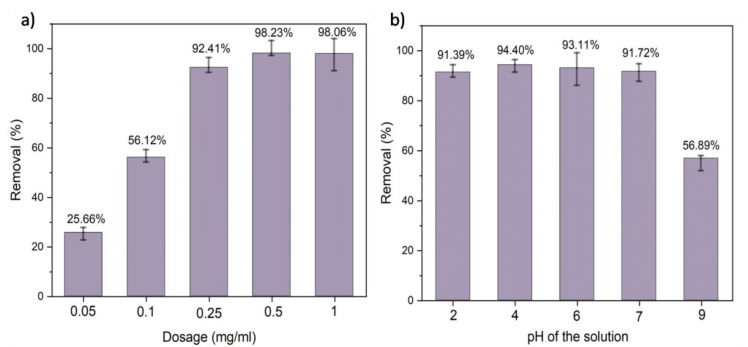
Removal (%) (**a**) at different dosage of adsorbent (pH = 6, adsorbent dosage = 0.25 mg/mL to 1 mg/mL, Cr concentration = 10 ppm, contact time = 30 min) and (**b**) at different pH values of the aqueous chromium solution (adsorbent dosage = 0.25 mg/mL, Cr concentration = 10 ppm, contact time = 30 min).

To optimize the ideal dosage of adsorbent for the efficient removal of Cr from its aqueous solution, the effect of dosage was investigated. Different dosages of adsorbent (CFC50) that is 0.05, 0.1, 0.25, 0.5, and 1 mg/mL were explored for the removal of aqueous solution of Cr(VI) under similar conditions. Figure 5a shows that the adsorption efficiency of CFC50 increased as we increased its dosage from 0.05 to 0.5 mg/mL.The increase in %efficiency is due to the availability of more active sites as the adsorbent dosage is increassed. The adsorption efficiency changed from 98.23% to 98.06% for an increase in adsorbent dosage from 0.5 to 1 mg/mL. This observation indicating saturation of adsorption by the sample is in agreement with the fact that the adsorption process follows the Langmuir isotherm model as inferred from isothermal analysis. After reaching the optimum dosage, the equilibrium was attained between the adsorbate and the adsorbent at a particular condition. Hence, the % efficiency also became saturated. Similar results were reported by Malhotra et al., (2018) where they found a decrease in adsorption with increase in dosage of adsorbent after attaining the optimum dosage condition [55]. It has been reported that increasing adsorbent dosage leads to the overcrowding of particles which led to a decrease in the adsorption performance. Keeping in mind the economic point of view, all the adsorption studies were performed taking 0.25 mg/mL as the optimum adsorbent dosage.

### 5.6. Effect of pH

**Figure 6:**
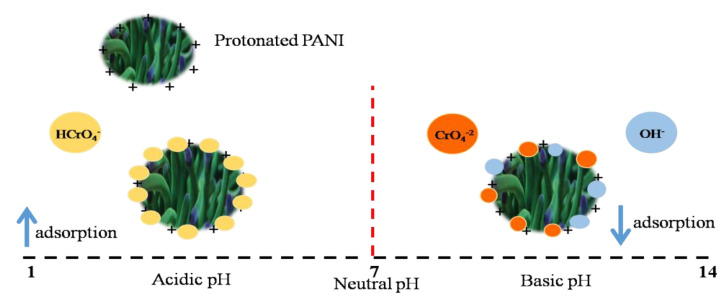
Schematic showing the mechanism of adsorption of chromium at acidic and basic pH ranges.

The pH of the solution plays a vital role during the adsorption studies as it influences the adsorption mechanism. The effect of pH was analyzed by preparing Cr(VI) solutions with different pH values, i.e., 2, 4, 7, and 9. The adsorbent dosage was kept at 0.25 mg/mL in 10 ppm of chromium solution with a contact time of 30 min. The pH of the aqueous solution of Cr(VI) was found to be 6. It was observed that the adsorbent exhibited superior performance in acidic conditions as shown in Figure 5b. Removal efficiencies of 91.39%, 94.40%, and 93.11% were observed at pH 2, 4, and 6 respectively. On the contrary, a reduction in removal (%) was observed at higher pH values. With the increase in pH from 6 to 9, a reduction in removal (%) from 93.11% to 56.89% by CFC50 was observed. From the pH study performed, it is concluded that the optimum pH for the adsorption of Cr(VI) is acidic. At acidic pH, Cr(VI) species generally exist as HCrO_4_^™^ ions which increase their electrostatic attraction with the highly protonated polymer composite. Similar findings showing the protonation of PANI were reported by Sulimenko et al. and Stejskal et al. [56,57]. This allows additional removal of chromium in acidic media. At basic pH, HCrO_4_^™^ becomes converted into CrO_4_^2-^. This creates competition between OH⁻ and CrO_4_^2™^ ions to become adsorbed on the surface of the adsorbent resulting in lower adsorption of chromium. The entire mechanism of the interaction of the adsorbent with the analyte (Cr(VI)) over the acidic and basic pH range is represented in Figure 6.

On comparing the results obtained from the present study with other similar research work as summarized in Table 5, we can conclude that the composites prepared in this study showed better results in terms of adsorption efficiency, adsorbent dosage, contact time, and utilizing low-cost adsorbent.

### 5.7. Adsorption Mechanism

Every adsorption process has a unique mechanism. The mechanism of adsorption depends on the interaction of adsorbent and adsorbate. The interaction is influenced by multiple factors such as surface charge, surface area, the nature of the analyte, and functional groups present on the surface of the adsorbent, etc. The understanding of the mechanism and its dependence on surface characterization are very important. Mechanism details enable us to modify the adsorbent and improve its performance. From the analysis of the adsorption process and characterization of the adsorbent, the proposed mechanism is as follows. The adsorption of chromium by CF-PANI composites followed the PSO kinetics, indicating that the adsorption by these materials occurs by chemisorption [69]. There is a strong attraction between positively charged amine functional groups (–NH–, –NH_2_) present on the surface of the polymer (confirmed from the IR spectrum of PANI) and negatively charged HCrO_4_⁻^-^ in acidic medium. Owing to this, it can be anticipated that electrostatic interaction could be a possible mechanism to explain the adsorption of Cr(VI) in the present study. As inferred from the IR results the presence of oxygen and nitrogen containing functional groups also help in the binding of Cr(VI) as mentioned in the results obtained from FTIR analysis [42]. Hence the preparation of composites provides more active sites for the enhanced removal of Cr(VI) due to dispersion of PANI on CF. Similar mechanisms have been reported by Deng et al. (2015) and Chigondo et al. (2019) for chromium adsorption using Polyethylenimine-modified fungal biomass and Magnetic arginine-functionalized polypyrrole, respectively [70,71].

## 6. Conclusions

Composites of CF and PANI were prepared with different loadings of PANI (15, 25, 50, and 75 *w*/*w%*). The composites were characterized using FTIR, FE-SEM, and FTIR spectroscopy for their surface and functional analysis. Subsequently, the prepared composites were used as adsorbents (0.25 mg/mL) for the removal of hexavalent chromium (10 mg L^−1^) from its aqueous solution. Our findings demonstrated that the CFC50 composite was most effective for the removal of Cr(VI) exhibiting 93.11% adsorption in 30 min. It showed an enhanced removal capacity as compared with pristine PANI (88.41%). The kinetics studies indicated towards the pseudo second order (R^2^ = 0.9937) of the removal process. The effects of adsorbent dosage, pH of the chromium and concentration of the chromium solution on the adsorption performance of CFC50 were also studied. The adsorption efficiency increased with increasing adsorbent dosages of CFC50. Moreover, from the pH studies it was inferred that the acidic pH is more suitable for the adsorption of Cr(VI) in its aqueous solution. This is due to the existence of Cr(VI) as a HCrO_4_^-^ ion in acidic medium which increases its electrostatic attraction with the highly protonated polymer composite. The adsorption of Cr(VI) by CFC50 was well described by the Elovich kinetic (R^2^ = 0.9625) and Langmuir isotherm models (R^2^ = 0.9888). The nature of adsorption was found to be monolayer and occurred via chemisorption. Thus, our study effectively displayed the suitability of the prepared CF-PANI composite in the treatment of Cr(VI). Development of CF-PANI composites results in enhanced adsorption performance of PANI for Cr(VI) removal and also is feasible considering the economic aspect in mind. This will contribute towards the removal of harmful pollutants and environmental mitigation which will open new doors in the field of adsorption. Moreover, field studies using the CF-PANI composites as an adsorbent for wastewater treatment should be considered in future. The CF-PANI composites evaluated in this study can be further up scaled and used in water purification systems as a filtration medium owing to their excellent performance as adsorbents.

## Figures and Tables

**Table 1:** Nomenclature and starting weight of the reagents for the preparation of composites.

Sample Code	CF (gm)	Weight of PANI (gm)	Weight of APS (gm)	Weight of Product (gm)
CFC15	0.5	0.075	0.231	0.34
CFC25	0.5	0.15	1.54	0.36
CFC50	0.5	0.5	1.54	0.81
CFC75	0.5	1.5	4.63	1.25

**Table 2:** Decomposition temperature and % weight loss with varying composition of PANI.

Sample Code	% Weight Loading of PANI (Theoretical)	Decomposition Temperature (T_d_)	% Weight Loss from TGA
CF	0	343	79.5
CFC15	15	396	79.9
CFC25	25	424	77.6
CFC50	50	440	78
CFC75	75	455	77
PANI	100	485	84.8

**Table 3:** The values of K and R^2^ of different kinetic model fittings for the adsorption of Cr(VI) onto the prepared samples.

Sample Code	Q_e_(mg/gm)	First Order		Second Order		Pseudo First Order		Pseudo Second Order		Elovich	Intra-Particle Diffusion	
		K	R^2^	K	R^2^	K	R^2^	K	R^2^	R^2^	K_d_	R^2^
PANI	35.36	0.06592	0.8460	0.0242	0.9547	0.1172	0.8846	0.012	0.9836	0.8390	4.254	0.7478
CFC15	7.15	0.00419	0.473	0.00043	0.4727	0.0175	0.3347	0.134	0.9362	0.0549	0.198	0.1022
CFC25	29.52	0.03436	0.7558	0.007	0.8755	0.0503	0.9768	0.023	0.9884	0.9182	2.143	0.9427
CFC50	37.24	0.0733	0.8733	0.0383	0.8765	0.0981	0.9100	0.020	0.9937	0.9625	2.725	0.9630
CFC75	33.04	0.04476	0.5923	0.0134	0.7085	0.0718	0.7314	0.065	0.9984	0.7985	1.253	0.7239

K_d_ = intraparticle diffusion constant.

**Table 4:** Value of R^2^ and different constants for the Freundlich, Langmuir, and Temkin isotherm models for the adsorption of different concentrations of Cr(VI) by CFC50.

Freundlich			Langmuir			Temkin	
R^2^	K_f_	n	R^2^	B	q_max_	R^2^	K_T_
0.9730	2.534	5.420	0.9888	36.630	2.4024	0.9867	1.586

Note: K_f_[(mg/g)(L/mg)^1/n^] = Freundlich adsorption capacity constant; n = Freundlich intensity parameter;b (L/g) = constant indicating affinity between an adsorbent and adsorbate;q_max_(mg/g) _=_ maximum saturated monolayer adsorption capacity of the adsorbent; K_T_ = Temkin isotherm constant.

**Table 5:** Summary of research papers on the removal of chromium using PANI and bio-waste based adsorbents.

Adsorbent	Dosage of Adsorbent (g/L)	Time	Removal (%)	Q_e_(mg/g)	Ref.
Rice husk ash—Ppy—PANI	0.8	300 min	98%		[26]
Polypyrole-calcium rectorite composite	1	-		714.29	[58]
Metal-organic framework-alginate beads	50	-	98%		[59]
PANI—jute	2	180 min		62.9	[27]
PANI—silica	0.8	430 min	193.85%		[28]
Calcinated wheat bran	1	24 h		29.3	[60]
Tea leaves	-	24 h	84.5%		[61]
Palm kernel	0.5	45 min		19	[62]
Eggshell powder	125	120 min	60.96%		[63]
PANI—sugarcane bagasse	1	100 min		35.2	[64]
CoFe(2)O_4—_PANI	0.5	14 min		103.11	[65]
Arginine doped PANI—walnut shell	0.3	3 h	99%		[66]
Pomegranate peels—Ppy—PANI	10	90 min	95.35%		[67]
Sugarcane bagasse Oil cake Maize corn	20	60 min	92% 97% 62%		[68]
Coconut fiber-polyaniline composite	0.25	30 min	93.11%	37.24	Present study

## Supplementary Materials

The following supporting information can be downloaded at: https://www.mdpi.com/article/10.3390/polym14204264/s1, Figure S1. Schematicflow for the synthesis of (a) PANI, (b) CF-PANI composites, and (c) the digital images of the prepared samples; Figure S2. UV-Vis absorbance spectra for the kinetic studies at different time intervals of time (a) PANI (b) CFC15 (c) CFC25 (d) CFC50 (e) CFC75(pH = 6, adsorbent dosage = 0.25 mg/mL, Cr concentration = 10 ppm, total contact time = 30 min) (f) Absorbance (at λmax = 352 nm) vs. time of prepared composites for Cr(VI); Figure S3. Freundlich, Langmuir, and Temkin isotherm models for different concentration of Cr(VI) by CFC50 (pH = 6, adsorbent dosage = 0.25 mg/mL, Cr concentration = 10 ppm to 50 ppm, contact time = 30 min); Table S1. Linear equation forms for different kinetic models; Table S2. Linear and non-linear equations for Freundlich, Langmuir, and Temkin isotherm models.
