# Supplementary material for: Kinetic and Isothermal Investigations on the Use of Low Cost Coconut Fiber-Polyaniline Composites for the Removal of Chromium from Wastewater
Source: Polymers (Basel). 2022 Oct 11;14(20):4264. doi: 10.3390/polym14204264 (PMC9610989; doi:10.3390/polym14204264)
Supplement: Supplementary file 1 [file polymers-14-04264-s001.zip › polymers-1927555-supplementary.pdf]

Kinetic and Isothermal Investigations on the Use of Low Cost Coconut Fiber-Polyaniline Composites for the Removal of Chromium from Wastewater

StutiJha[1], Rama Gaur [1,*], Syed Shahabuddin[1,*], Irfan Ahmad [2] and Nanthini Sridewi[3,*]

[1] Department of Chemistry, School of Technology, Pandit Deendayal Energy University, Knowledge Corridor, Raysan, Gandhinagar 382426, Gujarat, India.
[2] Department of Clinical Laboratory Sciences, College of Applied Medical Sciences, King Khalid University, Abha 61421, Saudi Arabia
[3] Department of Maritime Science and Technology, Faculty of Defence Science and Technology, National Defence University of Malaysia, Kuala Lumpur 57000, Malaysia
* Correspondence: rama.gaur@sot.pdpu.ac.in (R.G.); syedshahab.hyd@gmail.com (S.S.) or syed.shahabuddin@sot.pdpu.ac.in; nanthini@upnm.edu.my (N.S.); Tel.: +91-8585932338 (S.S.); +60-124-675-320 (N.S.)

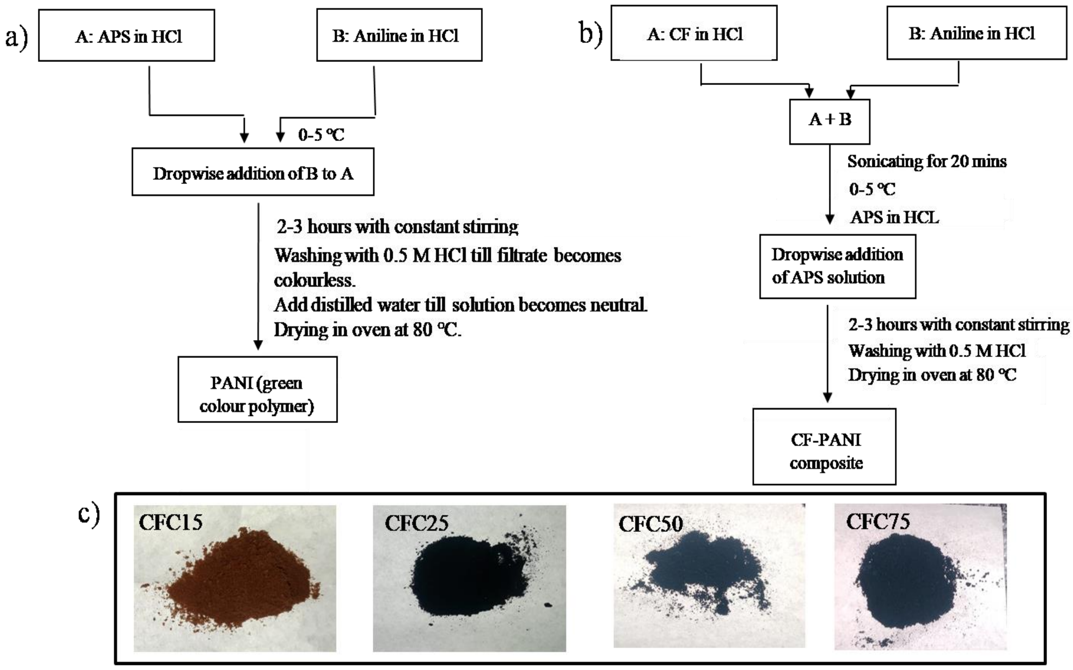

Figure S1. Schematicflow for the synthesis of a) PANI, b) CF-PANI composites, and c) the digital images of the prepared samples.

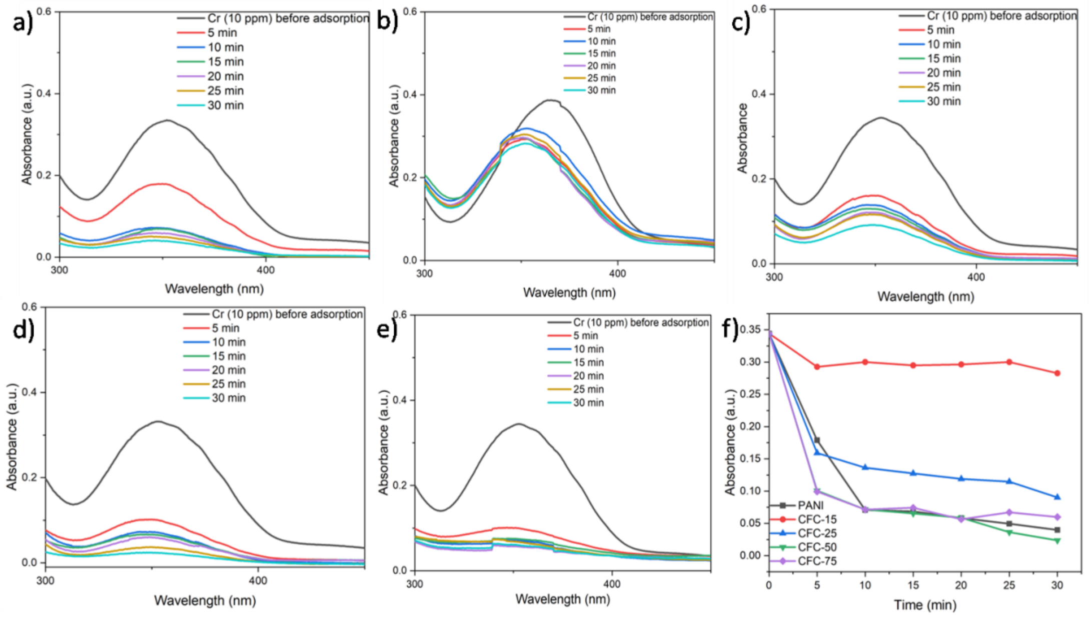

Figure S2. UV-Vis absorbance spectra for the kinetic studies at different time intervals of time a) PANI b) CFC15 c) CFC25 d) CFC50 e) CFC75(pH = 6, adsorbent dosage = 0.25 mg/ml, Cr concentration = 10 ppm, total contact time = 30 mins) f) Absorbance (at λ_{max} = 352 nm) vs. time of prepared composites for Cr(VI).

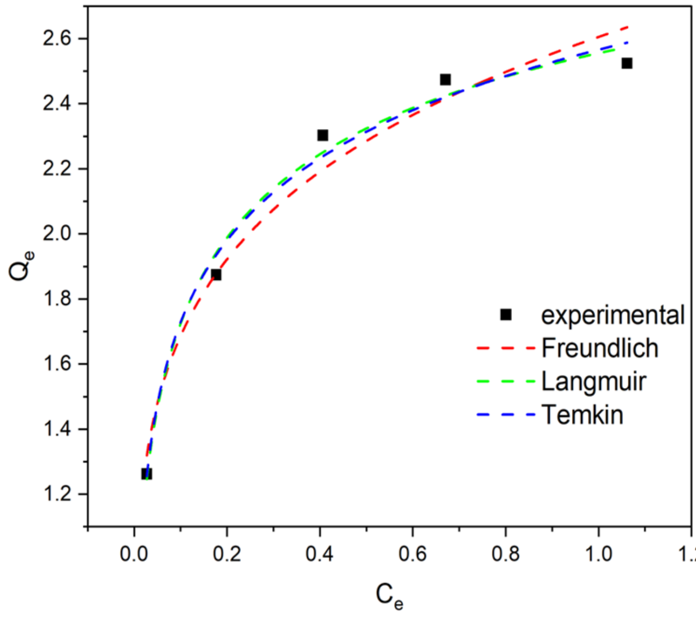

Figure S3. Freundlich, Langmuir, and Temkin isotherm models for different concentration of Cr(VI) by CFC50 (pH = 6, adsorbent dosage = 0.25 mg/ml, Cr concentration = 10 ppm to 50 ppm, contact time = 30 mins).

Table S1. Linear equation forms for different kinetic models.

Kinetic model	Linear equation
First order	$\ln C = -kt + \ln C_0$
Pseudo first order	$\ln(q_e - q_t) = \ln q_e - k_1 t$
Second order	$\frac{1}{C} = kt + \ln C_0$
Pseudo second order	$\frac{t}{q_t} = \frac{1}{k_2 q_e^2} + \frac{t}{q_e}$
Intraparticle diffusion	$q_t = k_d t^{0.5} + C$
Elovich model	$q_t = \frac{1}{\beta}\ln(\alpha\beta) + \frac{1}{\beta}\ln t$

NOTE: C = final concentration of adsorbate after adsorption; k = rate constant; t = time; C_o = initial concentration of the adsorbate before adsorption; q_e = amount of adsorption at equilibrium; $q_{t=}$ amount of adsorption at equilibrium; α = initial sorption rate constant; β = desorption constant.

Table S2. Linear and non-linear equations for Freundlich, Langmuir, and Temkin isotherm models.

Isotherm model	Linear form	Non-linear form
Freundlich isotherm	$\ln q_e = \ln K_f + n \ln C_e$	$q_e = K_f C_e^{\frac{1}{n}}$
Langmuir isotherm	$\frac{1}{q_e} = \frac{1}{q_{max} b C_e} + \frac{1}{q_{max} b}$	$q_e = \frac{q_{max} b C_e}{1 + bC_e}$
Temkin isotherm	$q_e = \frac{RT}{b_T}\ln K_T + \frac{RT}{b_T}\ln C_e$	$q_e = \frac{RT}{b_T}\ln(K_T C_e)$

NOTE: qe = amount of adsorption at equilibrium; C_e = concentration of adsorbate at equilibrium; k_f = Freundlich adsorption capacity constant; q_{max}= maximum monolayer adsorption capacity of adsorbent; b = constant related to affinity between adsorbent and adsorbate; R = gas constant; T = Temperature; b_T= Temkin constant related to heat of sorption; K_T = Temkin isotherm constant.
